# A Contrastive Study of Self-Assembly and Physical Blending Mechanism of TiO_2_ Blended Polyethersulfone Membranes for Enhanced Humic Acid Removal and Alleviation of Membrane Fouling

**DOI:** 10.3390/membranes12020162

**Published:** 2022-01-29

**Authors:** Abdul Latif Ahmad, Nuur Fahanis Che Lah, Nur Amelia Norzli, Wen Yu Pang

**Affiliations:** School of Chemical Engineering, Engineering Campus, Universiti Sains Malaysia, Nibong Tebal 14300, Malaysia; nuurfahanis@usm.my (N.F.C.L.); nuramelianorzli@gmail.com (N.A.N.); stanley930227@hotmail.com (W.Y.P.)

**Keywords:** polyethersulfone, antifouling, mixed matrix membrane, titanium dioxide, additives

## Abstract

In this study, membrane fabrication was achieved by two different methods: (i) self-assembly and (ii) physical blending of TiO_2_ in PES membrane for humic acid filtration. The TiO_2_ nanoparticles were self-assembled by using TBT as the precursor and pluronic F127 as triblock copolymers around the membrane pores. This was achieved by manipulating the hydrolysis and condensation reaction of TBT precursors during the non-solvent induced phase separation (NIPS) process. On the other hand, the TiO_2_ was physically blended as a comparison to the previous method. The characteristic of the membrane was analysed to explore the possibility of enhancing the membrane antifouling mechanism and the membrane flux. The membrane morphology, pore size, porosity, and contact angle were characterised. Both methods proved to be able to enhance the antifouling properties and flux performance. The HA rejection increased up to 95% with membrane flux 55.40 kg m^−2^ h^−1^. The rejection rate was not significantly improved for either method. However, the antifouling characteristic for the self-assembly TiO_2_/PES membrane was better than the physically blended membrane. This was found to be due to the high surface hydrophilicity of the MM membrane, which repelled the hydrophobic HA and consequently blocked the HA adsorption onto the surface.

## 1. Introduction

According to the World Health Organization (WHO), 785 million people live without even a basic drinking-water service, which includes 144 million people dependent on surface water. Unfortunately, at least 2 billion people use a contaminated drinking water source with chemicals and faeces, globally. Ineffective management of urban [1], industrial, and agricultural wastewater exposes individuals to dangerously contaminated or chemically polluted water resources. Thus, several technologies have been implemented and upgraded to address this problem throughout the decades. The effective development of high-rate separation technologies will provide not only a lower cost and small footprint but also offer communities favourable incentives to promote a sustainable lifestyle society [2]. In the drinking water industry, ultrafiltration (UF) membrane has been broadly used as it possesses the effectual ability to remove microorganisms, natural organic matter, particles, and turbidity compared to conventional filtration [3]. Significant effort and improvement has been carried out to remove humic acid (HA) via membrane filtration since the presence of HA in water can threaten life. HA has high hydrophobicity and will cause membrane fouling to occur frequently on the hydrophobic membrane [4]. However, the use of hydrophilic membrane material for HA removal has a high tendency to swell in water and lose mechanical strength. Ergo, the hydrophilicity and hydrophobicity of the material were manipulated to establish the best method with high membrane flux and minimize the fouling [5].

Polyethersulfone (PES) membrane has been extensively used in water filtration systems as a good separation membrane. To enhance the membrane hydrophilicity, a few methods have been implemented such as modification in bulk, surface, and blending [5]. One of the examples of bulk modifications for PES membrane is the sulfonation reaction. This reaction is an electrophilic reaction, which is part of an aromatic ring, usually, the hydrogen ion is replaced with hydroxysulfonyl radical or sulfonic acid group (-SO_3_H). However, the technique has proved difficult to carry out since the adjacent aromatic ring for electrophilic substitution was destroyed due to the sulfone linkage’s electron-withdrawing effect, which limited the membrane modification procedure [6]. Surface modification of PES membranes, on the other hand, has been established for a variety of applications, including addressing the fouling problem. One of the methods is protein grafting into the membrane. As a result, the hydrophilicity of the membrane surfaces have been shown to increase, lowering the contact angle [7].

The blending mechanism is the most extensively employed of these three modification methods since it has been demonstrated to be an effective approach for modifying porous filtration membranes by adding hydrophilic polymers or inorganic additives to improve permeability and antifouling properties [8]. In addition, the blending method is considered a convenient method in terms of operation, and only mild preparation conditions are needed [9]. Previous researchers have demonstrated that by adding hydrophilic additives with PES membrane, it can improve the filtration efficiency and performance of the membrane. Some of the hydrophilic additives that have been studied are polyvinylpyrrolidone (PVP), polyethylene glycol (PEG), cellulose phthalate (CAP), and sulfonated poly (arylene ether sulfide sulfoxide sulfone) (SPAESSS). In bovine serum albumin (BSA) filtration, to minimize the antifouling of the PES membrane, PES was blended with 2 wt% PVP of two different molecular weights (40 kDa and 360 kDa) [7]. Before adding the PVP, when conducting the testing of casted flat sheet with BSA, due to the existence of cake formation and BSA pore blockage, it has been shown that fouling is more serious, but by adding the PVP, the fouling problem was reduced. This happened because PVP has hydrophilic properties, which can prevent the cake formation from being excessive and reduce the pore blockage by BSA. Hence, it proves PVP improved the antifouling of the PES membrane, but the fouling problem still cannot be eliminated. Thus, researchers have included several other nanoparticles as potential substances to address the limitation. The photocatalytic technology of TiO_2_ has been selected as one of the promising nanoparticles based on the characteristic of hydrophilicity, stability, antibacterial properties, and low cost [10].

The key problem in large water treatment systems is in handling the slurry after the treatment for recovery and recycling of TiO_2_ nanoparticles. It was time-consuming and considered an expensive process. To solve this problem for its commercialization, several procedures and methods were developed to incorporate the TiO_2_ nanoparticles, which include layering on supporting substrate and physically blending. The blending of TiO_2_ with polymeric membrane has proven to facilitate wastewater treatment. However, the physical blending method was widely known to have the limitation of two factors: (i) aggregation of nanoparticles and (ii) the nanoparticles leached out from the membranes, which will harm the environment. In long-term operation, previous research shows that the self-assembly method features include it being an eco-friendly, human-friendly, and cost-effective separation tool in water separation systems compared to the physical blending method [11]. Thus, the self-assembly method has been preferred in membrane casting rather than the physical blending method.

Several studies have revealed varying TiO_2_ composite membrane performance subject to the synthesis and formulation of the composite membrane, and the wastewater characteristics. TiO_2_ has generally enhanced the hydrophilicity and fouling resistance of the mixed matrix membrane. For example, in a BSA filtration study of self-assembly of TiO_2_-PES membrane shows that the hydrophilicity of TiO_2_ nanoparticles could immobilize water molecules in the vicinity of PES membrane [11]. On the other hand, as the filtration was tested with humic acid, it was shown that TiO_2_ added slowed down the membrane saturation. The addition of in situ self-assembly TiO_2_ nanoparticles and F127 decreased the roughness of the PES membranes, which further lessened the organic fouling. Razmjou et al. reported good spreading of TiO_2_ nanoparticles in the membrane was achieved after both chemical and mechanical modifications of particles; therefore, less agglomeration was achieved and it lead to higher water flux [12,13]. This may be due to the increased porosity and pore size, and hydrophilicity of chemical modification. However, controlling the nanoparticles from leaching out remains a problem. This problem is usually correlated with the nanoparticle’s interaction with the polymer matrix. As a result, stabilizing the interaction between these two is preferred to ensure the membranes’ high efficiency. In an effort, the fouling evaluation of two precursors with both methods in the same condition was performed. It would provide a favorable indication of the applicability of TiO_2_ self-assembly and physical blending in a real ultrafiltration application of a PES/PVP MMM while also monitoring the membranes’ antifouling capabilities for HA separation fairly.

## 2. Materials and Methods

### 2.1. Materials

BASF (Ludwigshafen, Germany) supplied the PES Ultrason E6020 P. Acetic acid, N.N-dimethylacetamide (DMAc), PVP, TiO_2_ nanoparticles (21 nm), HA, Pluronic F127, and Titanium (IV) butoxide (TBT) were obtained from Sigma–Aldrich, USA. Permeation test and HA solution were prepared by using deionized water. Whereas distilled water was employed in membrane washing steps and throughout the phase inversion process.

### 2.2. Membrane Formulation and Casting Method

The dope solution was formulated by the self-assembly and physical blending method. For the self-assembly method, acetic acid was added to the DMAc solution with a 1:36.5 volume ratio. The solution was mixed with Pluronic F127. The solution was stirred at 500 rpm for 1 h at room temperature. The dropwise technique was used for TBT addition to the solution and the solution was sonicated for 1 h until the mixture became transparent. Subsequently, PVP was added and continuously stirred for another 1 h at room temperature. Next, PES flakes were added and stirred at 500 rpm and the temperature of 60 °C for 24 h. To eliminate the bubbles entrapped in the mixture, a 1-h degassing step was added by immersing the dope solution in an ultrasonic bath. The physical blending of TiO_2_ followed the same procedure with a minus of Pluronic F127 and TBT addition in the solution. The details for the dope solution composition are listed in Table 1.

The non-solvent induced phase inversion (NIPS) method at ambient temperature was used to prepare the fabrication of membranes. The dope solution was cast with a 200-micron thickness. After the membrane was exposed to air for 10 s, it was quickly transferred and immersed into a coagulant bath of distilled water that act as a non-solvent for 24 h for the complete solidification process. Before the drying process, the membrane was rinsed again with distilled water to assure the residual solvent was removed entirely. Lastly, the membrane was left dried for 3 days at ambient temperature testing and characterisation was conducted.

### 2.3. Membrane Characterisation

Using attenuated total reflection-Fourier transform infrared spectroscopy (Thermo Scientific Nicolet Nexus 670, Massachusetts, MA, USA), the chemical functional groups of the membrane were assessed. After layering with gold using a sputter coater (quorum SC7620), the surface and cross-sectional membrane morphology were examined using a scanning electron microscope, SEM (Hitachi TM 3000 Tabletop, Tokyo, Japan). Using energy-dispersive X-ray spectroscopy, EDX, the presence and dispersion of TiO_2_ in the membrane matrix were confirmed. Sessile drop contact angle measurements (Rame Hart, Succasunna, United State) were used to determine the membrane surface hydrophilicity.

The rheological property was measured in terms of the dope viscosity quantified utilizing Brookfield digital Rheometer (Model DV-III, Toronto, ON, Canada). Dry-wet weight measurement was used to determine membrane porosity [14]. The porosity can be analysed using Equation (1):

(1) $$\varepsilon \left(\%\right)=\frac{\left[\left(W_{w}-W_{d}\right)/\rho_{w}\right]}{\left[\left(W_{w}-W_{d}\right)/\rho_{w}\right]+W_{d}/\rho_{p}}\times 100\%$$

where *ε* is the porosity of membrane, *W_w_* is the weight of wet membrane (g), *W_d_* is the weight of dry membrane (g), ρ*_w_* is the density of water [1.00 g/cm^3^], and ρ*_p_* is the density of polymer [1.37 g/cm^3^].

The surface roughness of the membranes was analysed by atomic force microscopy (AFM, SPA400 SII Technology, Tokyo, Japan). The prepared membranes were cut to approximately 1 cm^2^ and glued on the glass plate and scanned at the area of 5 µm × 5 µm. Roughness parameters, such as the mean roughness (R_a_), the standard deviation of the height in the scanned area (R_q_), and the mean difference in height between the highest peaks and five lowest valleys (R_z_), were obtained. The average value of at least three random locations on the membrane surfaces was reported to minimize the experimental error.

The TGA 7 Thermogravimetric Analyzer (Perkin Elmer, Ohio, OH, USA) was used in the thermal resistance analysis for each sample. TGA analysis was determined under atmosphere air in heating rate from 30 °C to 850 °C, at ramping of 20 °C min^−1^. When the temperature reached 850 °C, heating was stopped, and the profile of significant weight loss was recorded. The content of the MM flat sheet membrane was evaluated from the residual weight.

### 2.4. Assessment of the Filtration Performance

**Figure 1:**
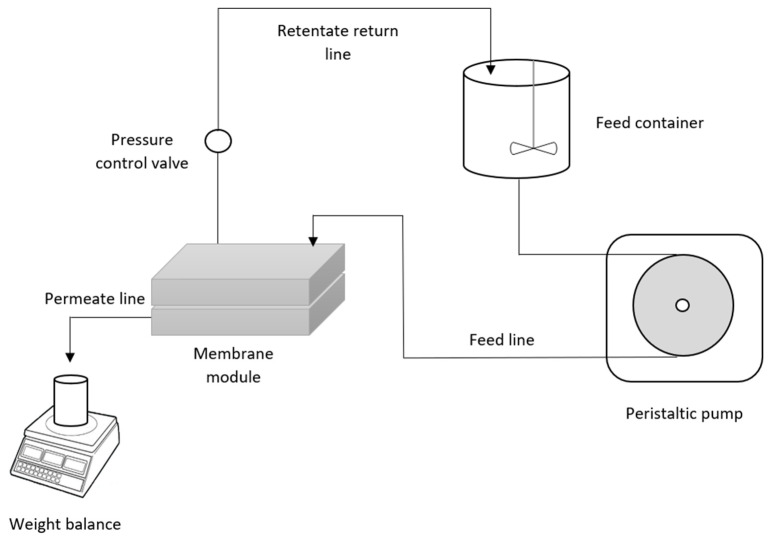
Schematic diagram of the permeation rig.

For the performance and antifouling test of the membrane, the filtration performance setup was carried out as illustrated schematically in Figure 1 with a 50 mg L^−1^ HA concentration. The feed solution concentration was chosen to assure fouling to examine the membrane’s antifouling capabilities while being within the typical range utilized by other studies [15,16,17]. Pure water was used for membrane compression at 1.5 bar for 20 min. The performance test was completed at a 1 bar for a duration of 1 h. A standard equation was used in calculating the initial pure water flux by applying Equation (2):

(2) $$J_{WF}=\frac{V}{A_{m}t}$$

here *J_WF_* is the pure water flux (kg m^−2^ h^−1^), *V* is the volume of permeate (L), *A_m_* is the effective filtration area (m^2^), and *t* is the measurement time (h).

The permeation test was first conducted by using the distilled water to collect the pure water flux at 1 bar. The distilled water was switched with the HA solution for the wastewater permeation study. After an hour of filtering performance, the concentration of both the feed and permeate solutions was measured using a UV–vis spectrophotometer (Cary 60) set to 254 nm and a 10 mm quartz cuvette. The fouling resistance of MMM was evaluated by calculating the relative flux reduction (*RFR*) using Equation (3):

(3) $$RFR\left(\%\right)=\left(1-\frac{J_{HA}}{J_{WF}}\right)\times 100\%$$

where *J_HA_* is the HA solution permeate flux (kg m^−2^ h^−1^) and *J_WF_* is the pure water flux (kg m^−2^ h^−1^). Consequently, the membrane cleaning was conducted by using distilled water to remove the foulant from the membrane surfaces for 15 min. The flux recovery ratio (*FRR*) of the membrane was calculated by using Equation (4):

(4) $$FRR\left(\%\right)=\frac{J_{WF2}}{J_{WF}}\times 100\%$$

where *J_WF2_* is the pure water flux after the washing step (kg m^−2^ h^−1^).

### 2.5. Analysis of Fouling Resistance

Fouling is generally classified based on several types of resistance throughout filtration. Darcy’s law is widely used in calculating the fouling resistance (refer to Equation (5)).

(5) $$J_{WF}=\frac{TMP}{\mu R_{t}}$$

where *TMP* is the transmembrane pressure (Pa), *μ* is the permeate viscosity (Pa.s) and *R_t_* is the total resistance of the membrane (cm^−1^). The *R_t_* can be derived into Equation (6):

(6) $$R_{t}=R_{m}+R_{f}=R_{m}+R_{r}+R_{ir}$$

where *R_m_*, *R_f_*, *R_r_*, and *R_ir_* are the membrane intrinsic resistance, resistance instigated by fouling, reversible, and irreversible fouling resistance, respectively. The membrane will only have its intrinsic resistance when there is no fouling occurred. Therefore, Equation (5) can be rewritten and rearranged as Equation (7):

(7) $$R_{m}=\frac{TMP}{\mu J_{WF}}$$

In real cases, the term *R_f_* would involve two factors: (i) fouling and (ii) concentration polarization effect. However, considering the difficulty to differentiate them, these two effects will be combined in this study. A pure water flux after membrane cleaning would imply the addition of *R_ir_* on top of *R_m_* for the membrane as the fouled membrane that goes through the cleaning process preserves the irreversible fouling. The model of the resistance is summarised in the Equations (8)–(10).

(8) $$R_{f}=\frac{TMP}{\mu J_{HA}}-R_{m}$$

(9) $$R_{ir}=\frac{TMP}{\mu J_{WF2}}-R_{m}$$

(10) $$R_{r}=R_{f}-R_{ir}$$

### 2.6. Fouling Mechanism Recognition

For deeper investigation of the foulant resistance, the fouling mechanism was identified by using the four models of Hermia [18] that describe the fouling mechanisms under constant pressure and combination models by Bolton et al. [19]. The latter combined two different individual fouling mechanisms with consideration of possibility having clogging mechanism combinations. The analytical expressions are summarised in Table 2. The cake formation that generates additional resistance occurred as the particles present in the wastewater were larger than the membrane pore itself. The resistances created were different based on the pore-clogging mechanism. The complete pore-blocking model presumes that each particle that reaches the membrane surface will clog the pore completely. Whereas the intermediate clogging has the probability of two conditions, which is the open pore blockage or particles build-up on formerly clogged pores. On the other hand, the standard blocking model occurred when the tinier particles adsorb onto the inner walls of the membrane pores by reducing the pore volume and enhancing the hydraulic resistance. The models were fitted by using Sigma plot 12 software (Systat Software Inc, Krakow, Poland).

## 3. Results

### 3.1. Membrane Morphology and Surface Analysis

**Figure 2:**
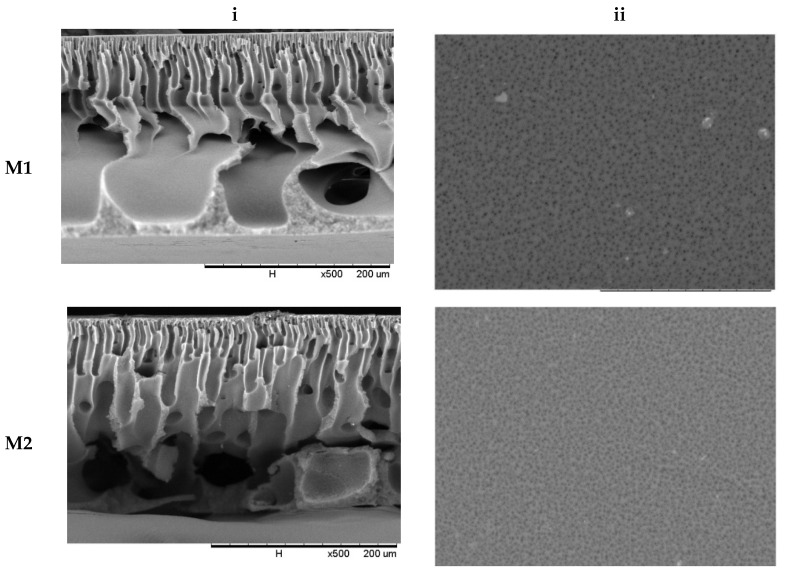

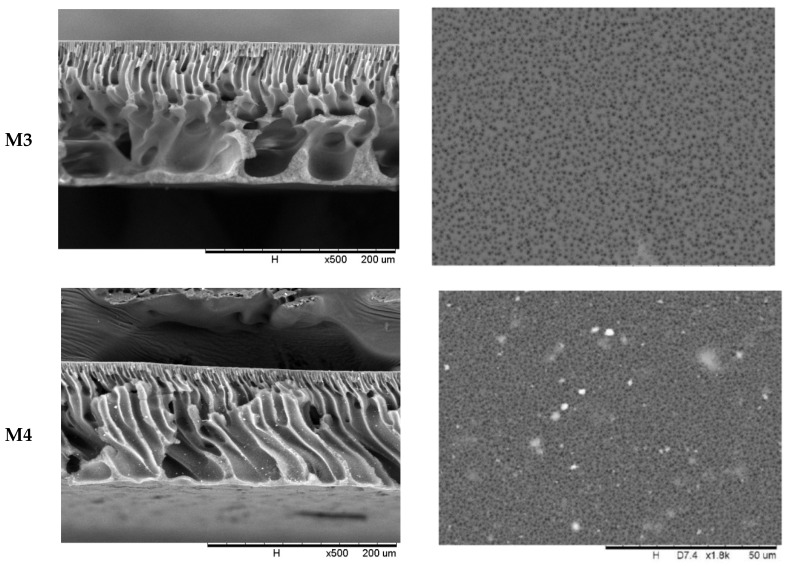
Membrane morphology of M1, M2, M3, and M4 membrane (**i**) cross-section at 500× magnification and (**ii**) outer surface at 1800× magnification.

The characterisation of the membrane morphology was analysed using SEM analysis. In cross-sectional morphology, Figure 2 displays two separate layers, which consist of a membrane with a thin and dense top layer supported by an anisotropic substructure. The top skin layer serves as a separating layer, while the bottom layer reinforces the membrane structure mechanically. The phase inversion that happened as the membrane was submerged in the coagulation water resulted in the anisotropic structure of the membrane morphology. It is well known that the presence of nanoparticles in the outer layer, which is responsible for the separation process, improves membrane separation capacity and performance. The surface morphology of the MMM is similar to a pristine membrane. However, the trace of TiO_2_ can be spotted only on top of the physical blend membrane (M4). It shows that no alteration of surface morphology was observed with the addition of TBT or F127.

**Figure 3:**
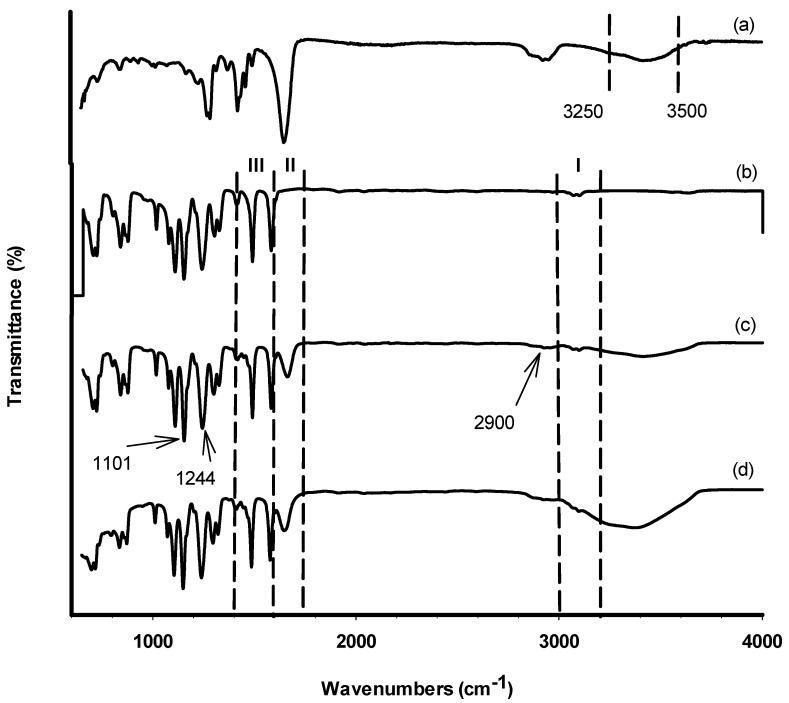
FTIR spectra (600–4000 cm^−1^) of (**a**) PVP, (**b**) PES, (**c**) blank membrane, and (**d**) M3.

Since the functional groups on the membrane’s surface are not visible through SEM, the TiO_2_ for self-assembly MMM was validated by utilising FTIR spectroscopy to characterise them. To identify the attribution of the material of the functional groups on the membrane’s surfaces, FTIR analysis for raw PES membrane has also been conducted. The FTIR spectra of the M3 membrane sample are shown in Figure 3. Several peaks have been observed in confirmation of the TiO_2_ and PVP existence embedded in the membrane structure. The C=O stretching of the PVP spectrum is represented by the absorption band at 1681 cm^−1^, whereas the O–H stretching vibration is represented by the band between 3250 and 3500 cm^−1^ [20,21]. In general, the inclusion of a -OH group improves the membrane’s anti-fouling capabilities by increasing its hydrophilicity [22]. The difficulty that frequently developed in membranes when PVP was utilised was primarily caused by the fact that it was easily dissolved from the dope solution during the phase inversion processes [23]. However, the higher affinity between the PES and PVP may have instigated some influence that further retains PVP from completely dissolving in the coagulation bath. Due to the sheer presence of a non-ionic surfactant in the PES membrane solution, the functional group of -CH_3_ methyl group may be identified at 2900 cm^−1^. The peak for F127 was detected at the range wavelength of 1200–1400 cm^−1^.

A difference between the spectrum of the PES polymer in Figure 3b and the synthesized membranes in Figure 3c,d can be observed in region II. Owing to the addition of PVP, the synthesized membranes showed an absorption band at 1681 cm^−1^ that was characteristic of PVP C=O stretching as previously seen in Figure 3a. Table 3 shows the results of the EDX study, which shows the core elements on the membrane’s top surface. Carbon (C) and oxygen (O) were detected in the membrane because of PES and PVP [14]. Only M2 membrane was not detected with Ti component which confirmed there is no Ti component in the membrane compared to the other three samples. However, the SEM surface images for the M1 and M3 membrane did not show any sign of TiO_2_ particles on the surface even though it was detected through the EDX analysis. This could be related to the non-uniform distribution of TiO_2_ nanoparticles on the membrane surface, as the TiO_2_ nanoparticles could not be observed when characterisation was performed on a specific region of the membrane surface.

Due to the adjustment in the component of casting solution and different compositions of component, the cross-section of different membranes presented a change in microstructure. The difference in the diffusion rates of the solvent and non-solvent induced the exchange process in the TiO_2_ self-assembly methods of the M1 membrane, resulting in the development of a finger-like structure at the membrane support layer, while hydrolysis and TBT condensation occurred to form the hydrophilicity Ti-OH groups [11]. As a result, an enlarged finger-like structure was formed. The macrovoid dimension increases as TiO_2_ is formed, which could be linked to the nanoparticle interference effect during the phase inversion process. The interfacial stress between the polymer and the nanoparticles causes the polymer phase to shrink during the demixing process, resulting in the formation of interfacial holes [24]. Due to the addition of Pluronic F127 to the M2 membrane, the slender shape holes at the support layer are generated from finger-like pores structure. During the NIPS process, the F127 addition reduces the solvent-non-solvent affinity while also increasing the non-solvent in-diffusion rate [25]. As a result, the transition of finger-like pores occurs. The process of forming different sizes of pores was affected by the miscibility between the coagulant and the surfactant itself. As a result, the introduction of a convenient surfactant can develop or limit the finger-like pores at the support layer [26].

The bottom layer of the M3 membrane shows a bigger finger-like pore structure compared to M4 membranes. By adding the nanoparticles of TiO_2_ and Pluronic F127, the hydrophilic feature of Ti-OH groups and PEO segment improved the demixing process of the membrane. The nanoparticles improve the smoothness of the structure and increase the number of membrane pores, while F127 accelerates the non-solvent in-diffusion rate [27], which improves the pores’ structure on the membranes. The SEM images also showed the same result as the theory. Table 4 shows that the higher value of viscosity slows down the precipitation, which resulted in a denser skin layer [28]. A denser skin layer can be observed through the cross-section of the membranes. In general, the strong interaction of solvent and polymer slowed down the coagulation as the viscosity increased. The diffusional exchange rate between DMAc and water in the membrane sublayer structure is influenced by both high polymer concentration and viscosity. Additionally, the precipitation rate at the sublayer level also slowed down as the diffusional exchange rate slowed down [29].

### 3.2. Membrane Hydrophilicity and Porosity

The membrane’s hydrophilicity is shown in Table 5. The decrease in water contact angle was thought to reflect an increase in membrane hydrophilicity. In comparison to the pristine membrane, the hydrophilicity of all mixed matrix membranes increases. It demonstrates that adding additives to membrane surfaces can boost their hydrophilicity. It confirmed the amelioration of membrane hydrophilicity by adding additives to membrane casting, which can perform a good performance in HA removal.

**Figure 4:**
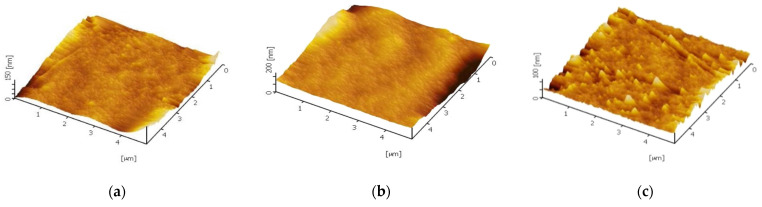
Three-dimensional AFM images for (**a**) Blank PES, (**b**) M3, and (**c**) M4.

The trend of static contact angle reduction can be clarified via two possibilities: (i) surface roughness increments and (ii) essential TiO_2_ nanoparticles hydrophilicity characteristic [30]. The pristine PES membrane shows hydrophilic characteristics with a contact angle to be found at 70.0°. The other membranes exhibit hydrophilic improvement for each configuration. This could be related to the surface roughness of the membrane when the nanoparticles are incorporated. Theoretically, the practicability between membrane hydrophilicity with surface roughness void when the intrinsic contact angle of materials is higher than 65° [31]. As the majority of the samples, in this case, demonstrate a lower contact angle than 65°, conclusively, the effect of roughness in enhancing hydrophilicity might be dominant. The membrane surface characteristic was analysed by AFM and the three-dimensional images of the membrane surfaces was depicted in Figure 4. The brightest area represented the highest point, and the dark regions indicated the valleys or membrane pores. The surface roughness of different methods is given in Table 6, and was calculated in an AFM scanning area of 5 µm × 5 µm. The mean roughness R_a_ of the membrane was decreased from 7.18 nm for pure PES membrane to 6.05 nm for self-assembly membrane whereas increase to 16.63 nm for physical blending membrane. Generally, smoother membrane surfaces have greater fouling resistance capability. Thus, the self-assembly TiO_2_-PES membrane shows the highest potential fouling resistance ability compared to blending TiO_2_-PES membrane. This will be further discussed in the next section. Additionally, the hydroxyl group present on TiO_2_ is supposed to assist in boosting the water droplet’s interaction with the membrane surface [32]. Consequently, the TiO_2_ integrated membrane matrix will have higher hydrophilicity characteristics in contrast to the pristine membrane.

The membrane porosity reported in Table 4 was consistent with the SEM images in Figure 2. By adding the nanoparticles of TiO_2_, the length of macrovoids was increased and produced a more granular pore wall. Meanwhile, the nanoparticles increased the porosity of the membrane by causing the macrovoid to develop in the upper portions of the membrane [12]. Moreover, the inclusion of TBT in the dope solution reduced the interaction of the polymer-solvent molecules. The demixing process speeds up the interchange rate of solvent and non-solvent. F127, on the other hand, reduces the affinity between solvent and non-solvent, resulting in an increase in the non-solvent in-diffusion rate during the NIPS process [11]. These results are in concordance with the membrane morphology in SEM analysis.

**Figure 5:**
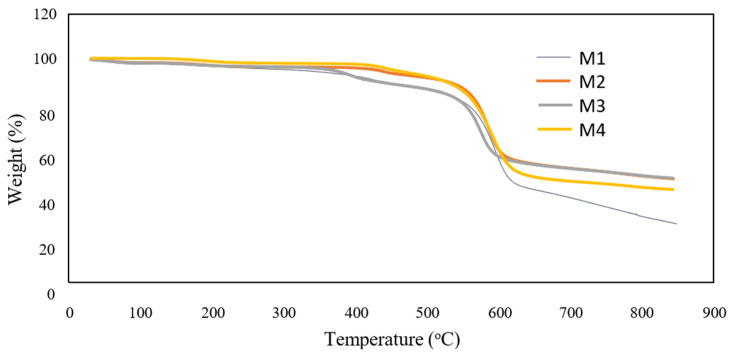
TGA analysis for the membranes.

The presence of TiO_2_ nanoparticles was also investigated by TGA analysis and the results are shown in Figure 5. It shows that the residual weight was higher with the addition of F127 compared to the M1. The result suggests that the self-assembly TiO_2_ nanoparticles are stabilised in the PES membrane matrix because of the presence of F127. The residual weight of M4 was lower compared to the residual weight of M3. The decomposition temperature for the membranes was around 600 °C. For TGA curves shown in Figure 4, the decomposition temperature weakly increased (around 10 °C) as a consequence of nanoparticles and Pluronic F127 incorporation into the polymer matrix. The rate of decomposition of the hybrid membrane slightly increased and indicates the polymer chains movement during heating is restricted with the formation of TiO_2_ particles [24]. A small increase was observed for the residual weight of the membranes at a temperature above 800 °C. The result shows M3 membrane has a good thermal resistance because, at 850 °C, it contained the highest residual weight.

### 3.3. Permeation Performance

Table 7 shows the effect of both techniques on pure water flow and the membranes’ HA rejection rate (HAR). As predicted, the filtration of the pristine membrane is lower than the flux for each mixed matrix membrane. The improvement of membrane hydrophilicity with TiO_2_ through self-assembly and blending methods in the mixed matrix membrane contribute to higher performance. A minimum value of 24.81 ± 1.73 L m^−2^ h^−1^ of pure water for the PES membrane was presented, whereas a maximum value of 59.83 ± 2.87 L m^−2^ h^−1^ was presented for M3 membrane out of all self-assembly membranes. In comparison, similar JHA can be observed between the M3 and M4 membranes. The high rejection rate may be attributed to the good dissemination of TiO_2_ nanoparticles. Well distribution of the particles minimized the agglomeration which prevent pore blocking. However, similar rejection rate and permeate flux by both M3 and M4 membrane proved that both methods can accentuate the TiO_2_ in the separation process. Thus, a deeper understanding of the system still needs to be established for a good comparison between these two methods.

### 3.4. Antifouling Properties Evaluation

Three primary phases of filtering with clean water and HA solution were used to further explain the antifouling performance of the pristine and mixed matrix membranes. These evaluations focus on the FRR and RFR. Both of these values are presented in Table 8. The FRR of a membrane was used to measure the antifouling properties, of which, higher FRR gives better antifouling properties. The membrane fouling was less severe when the RFR decreased as the RFR describes the severity of the membrane has been fouled [14]. Conclusively, a membrane with a low RFR value and high FRR value proved to be a good membrane with high antifouling properties. The MMM has a greater FRR than the pristine membrane, whereas the RFR value of the MMM was lower than that of the pristine membrane. Essentially, this membrane characteristic is correlated to membrane hydrophilicity. The hydrophilic groups in TiO_2_ and F127, which stimulated the production of hydrophilic water, formed a protective layer that repels the foulant on the membrane surface. Even though the M3 and M4 membranes have similar rejection rates and permeation, the M3 membrane has better antifouling properties than the M4 membrane because a hydration layer formed on the membrane surface by the hydrophilic groups in TiO_2_ [33] and PEO units in F127 weakened the interaction between organic pollutants and the membrane’s outer surface.

**Figure 6:**
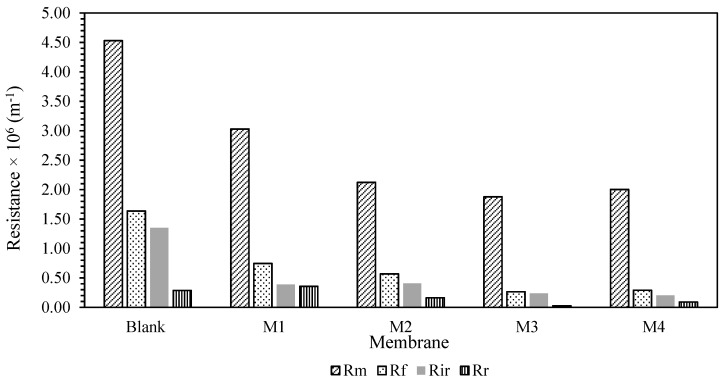
Filtration resistance of the membrane.

**Figure 7:**
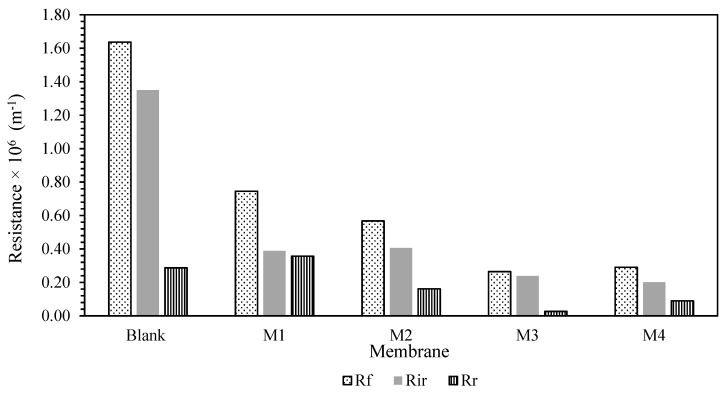
R_f_, R_r_, and R_ir_ of the membrane samples.

To gain a better understanding of the membrane fouling mechanism, Equations (7)–(10) were used to test a variety of resistances for membrane samples. The findings are shown in Figure 6. The fouling resistance (R_f_), reversible resistance (R_r_), and irreversible resistance (R_ir_) were also presented in Figure 7 for better clarification. It was presented that Rm was lowered with the blending of additive. This is in conjunction with the hydrophilicity characteristic of the additives, which reduces the membrane’s intrinsic resistance by accelerating the water flow through the membranes. However, due to the inadequate experimental time tested, the fouling resistance was observed to be quite low compared to Rm. Most of the resistance was mainly caused by the membrane resistance itself rather than the fouling phenomenon. Prior to long-term testing, a basic knowledge of the membrane’s antifouling capabilities had been established.

The R_ir_-HA fouling was decreased for all the MMM samples. A similar pattern was observed on R_r_. Nevertheless, the R_r_ for the M4 membrane was higher than the M3 membrane. This imposed resistance can be easily eliminated via proper cleaning for M4 membrane compared to self-assembly membrane despite having better antifouling properties with higher hydrophilicity characteristics. This will be an advantage in prolonging the operating lifetime of the membrane. However, the chances of contributors to irreversible fouling resistance are higher once a reversible cake is formed on the membrane surfaces. Despite this potential shortcoming, smaller overall foulant resistance R_f_ of the M3 membrane will be an extra point in antifouling properties due to its lower propensity of fouling. Overall, membrane resistance was noted as a dominant factor for membrane performance. However, in this case, different methods of membrane fabrication cannot enhance the membrane’s antifouling properties as there are slight/no changes in reversible resistance of the membrane. Nevertheless, the corporation of the TiO_2_ still provides an improvement in lowering the membrane resistance. Some modifications are needed to enhance the membrane antifouling properties for each fabrication method.

### 3.5. Fouling Mechanism Recognition

The classification of the fouling mechanism was completed for all the samples to deeply understand the fouling mechanism involved and observe the effect of both integrations of TiO_2_ methods on the membrane fouling. Both classical Hermia and Bolton models were used as a tool in classifying the fouling mechanism of the membrane. Table 9 shows the K and R^2^ values that were optimised for each model. Comparison of all the samples showed that no better adjustment was made even in the combined models used, compared to the Hermia model. In addition, the R^2^ of each sample is very close for Hermia models. This shows that there are no dominant factors or mechanisms in the flux change that can best describe these samples. However, the Hermia model constant for the M3 sample was higher than the constant model of the M4 sample. A similar observation was also obtained by Vela et al. [34] and Torkamanzadeh et al. [35] were for the ultrafiltration of polyethylene glycol and wastewater, R^2^ values for the four Hermia models were identical. Bowen et al. [36] described membrane blocking as consisting of four stages: total blocking, standard blocking, intermediate blocking, and cake development on the membrane surface. In our case, the coexistence of the blocking mechanism for each method proves there was no significant contribution of either method in reducing any specific fouling mechanism. The foulant resistance reduction in both methods compared to the blank membrane reduces as the TiO_2_ successfully impregnated in the membrane.

## 4. Conclusions

The TiO_2_ was assembled and successfully incorporated into PES/PVP for the membrane’s antifouling enhancement by two methods: (i) self-assembly and (ii) blending. Generally, both methods prove to help in enhancing the antifouling properties compared to the pristine membrane with a rejection rate up to 95.0% for sample M3 and 96.2% for sample M4. However, the presence of additives via self-assembly corporation (sample M3) was shown to be the membrane with the lowest fouling resistance, the highest permeate flow (55.40 ± 1.77 kg m^−2^ h^−1^), with high thermal resistance. With comparable rejection rate and permeate flux between these two methods, the self-assembly TiO_2_-PES membrane stands out with better characteristics in terms of antifouling properties compared to the blending TiO_2_-PES membrane. The antifouling performance of the developed membranes improved remarkably by using the self-assembly methods as the hydrophilicity of TiO_2_ and the F127 weakened the interaction of the foulant with the membrane surface. Overall, the mixed matrix membrane’s permeability and antifouling properties were improved by integrating the TiO_2_ with both methods without forgoing their separation efficiency.

## Figures and Tables

**Table 1:** Dope solution composition with different additives and mixing methods.

Membrane Sample	In Situ Corporation Composition				
	PES (g)	PVP (g)	TBT (mL)	F127 (g)	DMAc (mL)
Blank	17	5	0	0	76
M1	17	5	2	0	74
M2	17	5	0	1	75
M3	17	5	2	1	73
	Physical Blending				
	PES (g)	PVP (g)	TiO_2_ (g)		DMAc (mL)
M4	17	5	0.46		77.54

**Table 2:** Flux expressions of Hermia and Bolton Models.

Fouling Mode	Model	
	Hermia Model	
Cake formation (CF)	$J=\frac{J_{0}}{{\left(2K_{CF}J_{0}^{2}t+1\right)}^{1/2}}$	(11)
Intermediate blocking (IB)	$J=\frac{J_{0}}{K_{IB}J_{0}^{}t+1}$	(12)
Pore constriction (PC)	$J=\frac{4J_{0}}{{\left(K_{PC}J_{0}^{1/2}t+1\right)}^{2}}$	(13)
Complete blocking (CB)	$J=J_{0}exp\left(-K_{CB}t\right)$	(14)
	Bolton model	
Cake-complete (CF-CB)	$J=\frac{J_{0}}{K_{IB}J_{0}^{}t+1}exp\left(\left(\frac{-K_{CB}}{K_{CF}J_{0}^{2}}\right)\left(\sqrt{1+2K_{CF}J_{0}^{2}t}-1\right)\right)$	(15)
Cake-intermediate (CF-IB)	$J=\frac{J_{0}}{\sqrt{1+2K_{CF}J_{0}^{2}t}}\frac{1}{\left(1+\left(\frac{K_{IB}}{K_{CF}J_{0}}\right)\left(-1+\sqrt{2K_{CF}tJ_{0}^{2}+1}\right)\right)}$	(16)
Complete-standard (PC-CB)	$J=\frac{4J_{0}}{{\left(2+K_{PC}J_{0}^{}t\right)}^{2}}exp\left(\frac{-2K_{CB}t}{2+K_{PC}J_{0}^{}t}\right)$	(17)
Intermediate-standard (PC-IB)	$J=\frac{4J_{0}}{{\left(2+K_{PC}J_{0}^{}t\right)}^{2}\left(1+\frac{2K_{IB}J_{0}t}{2+K_{PC}J_{0}^{}t}\right)}$	(18)

**Table 3:** EDX study of the membrane samples.

Membrane	C (wt%)	O (wt%)	Ti (wt%)
M1	67.32	20.69	0.70
M2	65.55	22.76	-
M3	62.21	25.16	1.41
M4	65.16	20.97	1.33

**Table 4:** Viscosity of dope solution prepared.

Membrane	Viscosity (cP)
Blank	666.7 ± 2.1
M1	800 ± 1.7
M2	800 ± 2.2
M3	1100 ± 3.1
M4	850 ± 2.5

**Table 5:** Contact angle and porosity of the membrane samples.

Membrane Sample	Contact Angle (°)	Porosity, ε (%)
Blank	70.0	70.0
M1	61.2	75.7
M2	60.7	84.3
M3	43.9	89.4
M4	61.8	77.9

**Table 6:** AFM surface roughness values of the neat and hybrid membranes.

Membrane Sample	Surface Area (µm^2^)	Roughness		
		R_a_ (nm)	R_q_ (nm)	R_z_ (nm)
Blank	25.09 ± 0.04	7.18 ± 0.83	12.65 ± 0.56	85.64 ± 7.02
M3	25.11 ± 0.03	6.05 ± 0.51	8.97 ± 0.49	71.83 ± 2.09
M4	25.08 ± 0.01	16.63 ± 0.58	31.07 ± 1.38	141.2 ± 6.8

**Table 7:** Flux and rejection rate of the membrane samples.

Membrane	Pure Water Flux (L m^−2^ h^−1^)	Permeate Flux (L m^−2^ h^−1^)	HAR (%)
Blank	24.81 ± 1.73	20.30 ± 2.58	92.0
M1	37.10 ± 2.43	33.16 ± 3.21	92.3
M2	52.95 ± 3.48	46.92 ± 3.53	92.5
M3	59.83 ± 2.87	55.40 ± 1.77	95.0
M4	66.10 ± 4.78	55.15 ± 3.23	96.2

**Table 8:** Antifouling properties of the membrane samples.

Membrane	FRR (%)	RFR (%)
Pristine	77.04	29.92
M1	88.64	19.74
M2	83.94	21.10
M3	88.76	12.10
M4	82.93	26.55

**Table 9:** The R^2^ and estimated K values of the membrane samples for the Hermia and Bolton models.

	Hermia Models				Bolton Models			
	CF	IB	PC	CB	CF-CB	CF-IB	PC-CB	PC-IB
Blank	R^2^ = 0.9188	R^2^ = 0.9188	R^2^ = 0.9196	R^2^ = 0.9203	R^2^ = 0.9203	R^2^ = 0.9196	R^2^ = 0.9203	R^2^ = 0.9196
	K_CF_ = 4.8 × 10^−7^	K_IB_ = 1.92 × 10^−6^	K_PC_ = 1.87 × 10^−6^	K_CB_ = 0.0001	K_CB_ = 7.57 × 10^−28^ K_CF_ = 0.7808	K_CF_ = 7.95 × 10^−9^ K_IB_ = 4.67 × 10^−7^	K_PC_ = 4.78 × 10^−33^ K_CB_ = 0.0001	K_PC_ = 1.87 × 10^−6^ K_IB_ = 3.99 × 10^−13^
M1	R^2^ = 0.8911	R^2^ = 0.8911	R^2^ = 0.8925	R^2^ = 0.8937	R^2^ = 0.8937	R^2^ = 0.8925	R^2^ = 0.8937	R^2^ = 0.8925
	K_CF_ = 8.69 × 10^−7^	K_IB_ = 3.48 × 10^−6^	K_PC_ = 3.32 × 10^−6^	K_CB_ = 0.0001	K_CB_ = 4.04 × 10^−28^ K_CF_ = 0.1886	K_CF_ = 2.23 × 10^−8^ K_IB_ = 8.29 × 10^−7^	K_PC_ = 7.83 × 10^−22^ K_CB_ = 0.0001	K_PC_ = 3.32 × 10^−6^ K_IB_ = 1.46 × 10^−12^
M2	R^2^ = 0.6971	R^2^ = 0.6971	R^2^ = 0.7025	R^2^ = 0.7080	R^2^ = 0.7080	R^2^ = 0.7025	R^2^ = 0.7080	R^2^ = 0.7025
	K_CF_ = 3.38 × 10^−7^	K_IB_ = 1.35 × 10^−6^	K_PC_ = 1.33 × 10^−6^	K_CB_ = 6.52 × 10^−5^	K_CB_ = 1.46 × 10^−28^ K_CF_ = 0.9034	K_CF_ = 6.66 × 10^−9^ K_IB_ = 3.32 × 10^−7^	K_PC_ = 3.44 × 10^−21^ K_CB_ = 6.52 × 10^−5^	K_PC_ = 1.33 × 10^−6^ K_IB_ = 5.55 × 10^−14^
M3	R^2^ = 0.7514	R^2^ = 0.7514	R^2^ = 0.7666	R^2^ = 0.7820	R^2^ = 0.7820	R^2^ = 0.7666	R^2^ = 0.7820	R^2^ = 0.7666
	K_CF_ = 2.99 × 10^−6^	K_IB_ = 1.2 × 10^−5^	K_PC_ = 1.13 × 10^−5^	K_CB_ = 0.0003	K_CB_ = 2.02 × 10^−28^ K_CF_ = 0.1433	K_CF_ = 1.19 × 10^−7^ K_IB_ = 2.83 × 10^−6^	K_PC_ = 9.89 × 10^−21^ K_CB_ = 0.0003	K_PC_ = 1.13 × 10^−5^ K_IB_ = 1.03 × 10^−25^
M4	R^2^ = 0.9598	R^2^ = 0.9598	R^2^ = 0.9591	R^2^ = 0.9584	R^2^ = 0.9637	R^2^ = 0.9598	R^2^ = 0.9591	R^2^ = 0.9598
	K_CF_ = 2.16 × 10^−7^	K_IB_ = 8.64 × 10^−7^	K_PC_ = 8.46 × 10^−7^	K_CB_ = 5.73 × 10^−5^	K_CB_ = 4.32 × 10^−9^ K_CF_ = 3.6 × 10^−6^	K_CF_ = 1.56 × 10^−9^ K_IB_ = 4.08 × 10^−27^	K_PC_ = 8.46 × 10^−7^ K_CB_ = 8.75 × 10^−13^	K_PC_ = 1.1 × 10^−22^ K_IB_ = 8.64 × 10^−7^

## Data Availability

Data sharing is not applicable to this article.
